# Pharmacist and physician insight of vancomycin therapeutic drug-monitoring changes

**DOI:** 10.1017/ash.2022.282

**Published:** 2022-09-16

**Authors:** Eric R. Gregory, Lucy Stun, Matt J. Mason, Nicole M. Wilson, Kassem A. Hammoud

**Affiliations:** 1 Department of Pharmacy, The University of Kansas Health System, Kansas City, Kansas; 2 Division of Infectious Diseases, University of Kansas Medical Center, Kansas City, Kansas

## Abstract

The updated vancomycin guideline for treatment of serious methicillin-resistant *Staphylococcus aureus* infections prompted institutions to convert from trough to area-under-the-curve monitoring. The physician perception of the transition, coupled with that of pharmacists, was measured by pre- and postimplementation surveys. Both groups believed safety would be increased without efficacy changes.

Vancomycin, a glycopeptide antibiotic, was historically monitored using trough concentrations as a surrogate for its true pharmacokinetic-pharmacodynamic (PK-PD) index against *Staphylococcus aureus*, area under the curve (AUC).^1^ However, the recommended trough concentrations for treatment of serious methicillin-resistant *S. aureus* (MRSA) infections of 15–20 mg/L led to increased nephrotoxicity risk.^2^ As a result of these data, a revised guideline recommended replacing trough monitoring with AUC monitoring.^3^ In anticipation of this update, our institution transitioned to vancomycin AUC therapeutic drug monitoring (TDM), and we now report the results of pre- and postintervention surveys disseminated to pharmacists and physicians.

## Methods

The transition to AUC monitoring at our health system occurred in February 2020, where a pharmacist-to-manage (PTM) vancomycin protocol was already utilized in adult patients. Microsoft Excel (Redmond, WA) was used to calculate AUC with 2 serum drug concentrations, using first-order pharmacokinetic equations with a therapeutic AUC defined as 400–600 mg*h/L. Pharmacist and physician education was conducted, including both pharmacy and internal medicine department grand rounds presentations, an on-demand video lecture for pharmacists, pharmacist review sessions, an internal medicine resident core education presentation, and an infectious diseases division journal club presentation.

Survey questions were reviewed prior to dissemination by a subgroup of the antimicrobial stewardship program consisting of vancomycin superusers from various clinical service lines. To gauge the transition, pharmacists and select physicians at our institution were voluntarily surveyed using REDCap in a before-and-after manner; both were open for 4 weeks total.^4^ Physicians surveyed included those from the department of orthopedic surgery and sports medicine and the divisions of infectious diseases, pulmonary and critical care medicine, cardiology, hematologic malignancies and cellular therapeutics, and medical oncology. The preintervention survey was disseminated 4 weeks before the pharmacy department grand rounds presentation, which was also performed 1 year prior to the transition. The postintervention survey was distributed 1 year after implementation. E-mail reminders were sent to pharmacists and physicians 3 times.

Questions in the survey tool included efficacy and safety changes, time requirements, and more. Definitions of efficacy and safety were left to the discretion of the survey respondent. Statistical analyses were performed using SPSS Statistics version 25 software (IBM, Armonk, NY) with the 2-tailed Pearson χ^2^ test and the Fisher exact test where appropriate. Statistical significance was defined as *P* < .05. The survey was deemed a quality improvement initiative by the local institutional review board.

## Results

Of a possible 559 respondents, 128 individuals (90 pharmacists and 38 physicians) completed the full survey, for a response rate of 22.9%. Most were familiar with vancomycin trough monitoring and were comfortable adjusting doses based on such concentrations. However, 86.7% of respondents were uncomfortable changing the dose using AUC monitoring according to the preintervention survey (Table 1). One year after implementation, a significantly greater percentage of clinicians strongly agreed they were comfortable adjusting doses (2.7% vs 41.5%; *P* < .001). Workflow perceptions were no different between the 2 periods because most clinicians assumed that more serum drug concentrations and time were required for AUC monitoring: 68% versus 77% (*P* = .316) and 64% versus 58.5% (*P* = .293), respectively. However, a difference was noted in the estimated lowest trough value required to attain a therapeutic AUC (Table 1). No difference was observed in the perception of total daily dose requirements (*P* = .305). Regarding outcomes, fewer clinicians in the postintervention survey believed that the change to AUC monitoring resulted in increased efficacy (57.3% vs 32.1%; *P* = .002), whereas the vast majority thought safety would be increased in both surveys (80% vs 83%; *P* = .673).

**Table 1.** Preimplementation Survey Versus Postimplementation Survey Results

Survey Question	Preintervention Survey (n = 75),	Postintervention Survey (n = 53),	*P*
	No. (%)	No. (%)	Value
**Profession**			0.434
Pharmacist	55 (73.3)	35 (66.0)	
Physician	20 (26.7)	18 (34)	
**Estimated no. of patients using troughs in the past 6 mo**			<.001
0–25	18 (24)	37 (69.8)	
26–50	15 (20)	7 (13.2)	
51–75	15 (20)	7 (13.2)	
>75	27 (36)	2 (3.8)	
**Estimated no. of patients using AUC in the past 6 mo**			<.001
0–25	75 (100)	19 (35.8)	
26–50	0 (0)	14 (26.4)	
51–75	0 (0)	12 (22.6)	
>75	0 (0)	8 (15.1)	
**Estimated lowest trough value required to attain a therapeutic AUC**			0.001
At least 5–10 mg/L	8 (10.7)	19 (35.8)	
At least 11–15 mg/L	52 (69.3)	32 (60.4)	
At least 16–20 mg/L	14 (18.7)	2 (3.8)	
At least >20 mg/L	1 (1.3)	0 (0)	
**Difference in efficacy**			0.002
Decreased	4 (5.3)	0 (0)	
No difference	28 (37.3)	36 (67.9)	
Increased	43 (57.3)	17 (32.1)	
**Difference in safety**			0.673
Decreased	1 (1.3)	0 (0)	
No difference	14 (18.7)	9 (17)	
Increased	60 (80)	44 (83)	
**Time requirement for monitoring**			0.293
Less required	4 (5.3)	7 (13.2)	
No difference	23 (30.7)	15 (28.3)	
More required	48 (64)	31 (58.5)	
**Serum drug concentrations required for monitoring**			0.316
Less required	6 (8)	5 (9.4)	
No difference	18 (24)	7 (13.2)	
More required	51 (68)	41 (77.4)	
**Total daily dose required**			0.305
Less required	38 (50.7)	16 (30.2)	
No difference	30 (40)	34 (64.2)	
More required	7 (9.3)	3 (5.7)	
**Clinician is comfortable adjusting doses based on troughs**			0.646
Strongly disagree	2 (2.7)	3 (5.7)	
Disagree	3 (4.0)	4 (7.5)	
Agree	27 (36.0)	19 (35.8)	
Strongly agree	43 (57.3)	27 (50.9)	
**Clinician is comfortable adjusting doses based on AUC**			<.001
Strongly disagree	32 (42.7)	6 (11.3)	
Disagree	33 (44.0)	5 (9.4)	
Agree	8 (10.7)	20 (37.7)	
Strongly agree	2 (2.7)	22 (41.5)	

Note. AUC, area under the curve.

**Fig. 1.**
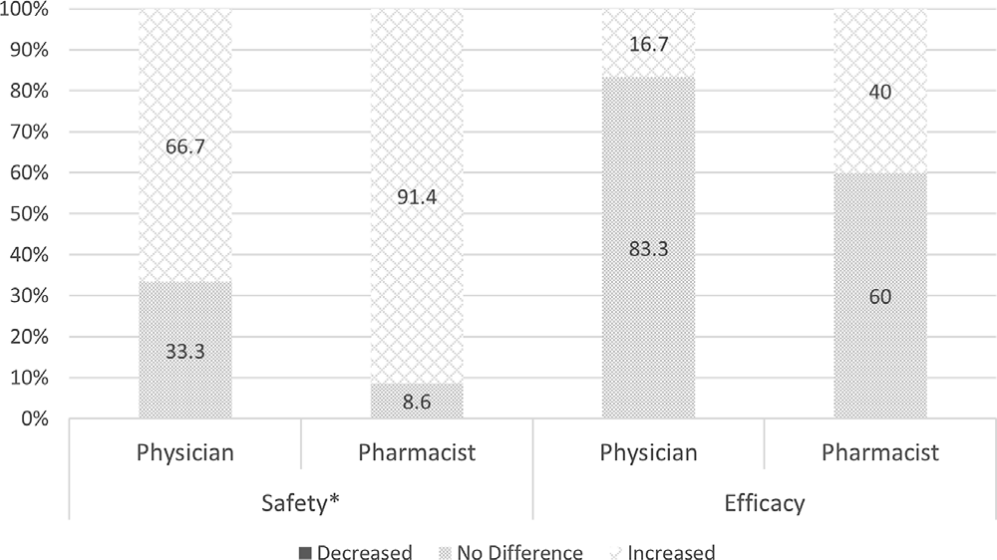
Postintervention clinical outcomes results comparing physicians to pharmacists. Efficacy, defined as clinical cure; safety, defined as reduced nephrotoxicity risk. An asterisk (*) denotes statistical significance, defined as *P* < .05.

Pharmacists and physicians in the postintervention survey group were compared to evaluate their respective perceptions of the changes after 1 year of AUC surveillance (Fig. 1). Just 2.9% pharmacists and 5.6% physicians believed a trough >15 mg/L was necessary to attain a therapeutic AUC (*P* = .321). Pharmacists were more comfortable adjusting doses based on both troughs and AUC (*P* < .001 and *P* = .001, respectively). Both groups perceived that more drug concentrations were necessary (*P* = .067), but pharmacists were far more likely to believe that more time was needed for AUC TDM compared to trough monitoring (80% vs 16.7%; *P* < .001). A numerical difference of the total daily dose was noted, with 74.3% of pharmacists believing that lower doses were sufficient to attain goal TDM markers compared to 44.4% of physicians (*P* = .074). Finally, a discordance was not observed related to any perceived changes in efficacy (*P* = .123); however, pharmacists were more likely to believe safety increased with the transition (91.4% vs 66.7%; *P* = .048).

## Discussion

Although vancomycin AUC TDM has been in practice for several years, this is the first known survey conducted to include both pharmacists and physicians. Specific questions were asked of the physicians with a goal of assessing the understanding and acceptance of pharmacist-to-manage vancomycin TDM. As a result of this survey, we hope to further inform the multidisciplinary practices and perception of the 2020 vancomycin guideline update for serious MRSA infections.

According to our survey results, an overall perception of increased safety was very apparent with the transition to AUC TDM. It was encouraging to note the perception of decreased toxicities not only after formal education was provided but also beforehand, as recognized in the pre- and postintervention surveys. Even though a statistically significant difference between pharmacists and physicians was identified in safety outcomes in the post implementation survey, we consider a mark of our successful education and implementation that both clinician groups believed increased safety would be seen due to the transition.

Evidence now firmly suggest that vancomycin is nephrotoxic due to acute tubulointerstitial damage.^5^ Although the most predictive pharmacokinetic measure of acute kidney injury is unclear at this time, it appears that AUC is much better correlated than trough concentration in both animal and human studies.^6,7^ According to prior survey results, other clinicians similarly appeared to notice these benefits firsthand. One pharmacist-only vancomycin AUC implementation survey was reported with 69% of respondents believing that safety was increased or slightly increased after the implementation of vancomycin AUC.^8^ Another pharmacist-only vancomycin AUC transition survey also found an overall increased perception of patient safety.^9^

Based on the idea that efficacy is unlikely to be impacted regardless of TDM method due to achieving AUC ≥ 400 mg*h/L in most cases with trough concentrations >15 mg/L, it is not surprising that the respondents in our postintervention survey believed that the change would not lead to increased efficacy.^10^ In fact, likely due in part to our education efforts, most of the postintervention survey respondents chose “no difference” in efficacy, whereas in the preintervention survey, 57.3% thought an increase in efficacy would be achieved.

A strength of this study is the review of the survey questions by a vancomycin superuser group prior to distribution. An anonymous answering method was used in the REDCap tool to increase the confidence of respondents. Response bias was a likely limitation of this study because respondents may have answered using anecdotal experience. For the preintervention survey, respondents were not able to be linked to those in the postintervention survey. Education bias was present in the postintervention survey. Finally, institutional data suggesting changes in efficacy and safety are currently lacking, limiting the perception of the clinician respondents.

Our pre– and post–vancomycin AUC implementation surveys, coupled with formalized education efforts, identified the popular belief among pharmacists and physicians that AUC monitoring leads to increased safety compared to using trough concentrations. Efficacy, on the other hand, was perceived to be unchanged. Further investigational efforts are necessary to validate these results.
